# Natural Film Based on Pectin and Allantoin for Wound Healing: Obtaining, Characterization, and Rat Model

**DOI:** 10.1155/2020/6897497

**Published:** 2020-10-17

**Authors:** Karen Zulema Meza Valle, Rosa A. Saucedo Acuña, Judith V. Ríos Arana, Naun Lobo, Carlos Rodriguez, Juan Carlos Cuevas-Gonzalez, Karla Lizette Tovar-Carrillo

**Affiliations:** ^1^Institute of Biomedical Sciences, Autonomous University of Ciudad Juarez, Juarez City, Chihuahua, Mexico; ^2^Departamento de Ingeniería en Nanotecnología, Universidad Tecnológica de Ciudad Juárez, Av. Univ. Tecnológica 3051 C.P. 32695 Cd. Juárez, Mexico; ^3^Grupo Evelsa, Hnos. Escobar 6150-2 Parque Ind. Omega C.P. 32340 Cd. Juárez, Mexico

## Abstract

**Introduction:**

In our days, several approaches reported the use of natural compounds in medical applications. Among them, pectin and allantoin are nontoxic, biocompatible, and biodegradable; however, its use for possible wound healing therapeutics is still limited. Pectin and allantoin have been applied in pharmaceutical industry and beauty cosmetic and could be also applied as scaffolds for tissue regeneration, wound healing, and so on. The aim of this study was to combine by the first time two natural ingredients to develop a new biomaterial to treat skin injuries in a rat model.

**Methods:**

For the hydrogel development, new synthesis parameters were established for the obtaining of the film such as temperature, mixing velocity and time, and drying temperatures as well. To enrich the film, the allantoin concentrations were set at 90 wt% and 100 wt% of pectin used. By in vivo assay, films were tested in wound healing in female Wistar rats, 190 ± 10 g in weight and 2 months aged.

**Results:**

The obtained films comprise 2 well-differentiated layers, one layer rich in allantoin, which will be the regenerative layer, and one rich in pectin, which will work as an antimicrobial and protective layer to the wound. These were characterized by swelling kinetics, Fourier transform of the infrared spectrum of absorption (FTIR) spectroscopy, and contact angle. The morphology and topography were determined by scanning electron microscopy (SEM) and confocal laser scanning microscopy (CLSM). In vivo assay showed remarkable reduce in a time period in a wound healing process when the film was used. The results show that the use of PA (Pectin-Allantoin) hydrogel reduces the total healing time by 25% approximately.

**Conclusions:**

Pectin-Allantoin (PA) film has potential use in medical applications as wound healing material promoting healthy tissue renewal.

## 1. Introduction

Hydrogels are polymeric networks crosslinked with great interest as biomaterials due to their great biocompatible properties [1–3]. Pectin is a biopolymer excellent to obtain hydrogels that can form matrices capable of absorbing and retaining hundreds of times their weight in water [2–8], due to precise carboxylic acid and methyl ester carboxylate groups which confers it a high polarity [8]. The use of this polysaccharide has been explored as a functional source for biomedical and pharmaceutical applications, such as the development of scaffolds for the release of drugs, wound healing, and tissue engineering, due to its natural gelling mechanism and high availability besides its nontoxic nature [7–11]. On the other hand, allantoin is a white powder highly used as a natural ingredient not only in cosmetic products but also in pharmaceutical products for skin diseases [6–8], since one of the main effects of this compound is the strong stimulation of cell proliferation and the reconstruction of intact granulation tissue, which justifies its use as an active ingredient unique in pharmaceutical preparations, which has been proven effective to treat ulcers, healing wounds, and burns[12–14].

Among the biomaterials used to fulfil extracellular matrix functions and provide protection during wound healing, promoting the regeneration and growth of new tissue [15], pectin became a remarkable option, which is a polysaccharide of plant origin, with excellent gelling properties and the ability to modulate the release of active molecules [16, 17]. These characteristics have been the basis for combining pectin with different chemicals, pharmaceutical and natural compounds with the aim of other properties such as regenerative and antibacterial effects. One natural compound with pharmaceutical, healing, analgesic, and antibacterial properties is allantoin [18]. This suggests that the combination of pectin and allantoin could have positive effects on the wound healing process [19].

For this work, we present the first results of the in vivo preclinical study of the hydrogel, specifically the rate of wound contraction in an animal model using Wistar rat. The results show that the use of PA (Pectin-Allantoin) hydrogel reduces the total healing time by 25% approximately. For this, preclinical studies of this proposal continue with the aim to reach commercial distribution, improving the quality of life and patient care.

## 2. Materials and Methods

### 2.1. Collection of Samples

The materials for this project were acquired as follows: glutaraldehyde (GA) and hydrochloric acid (HCL), and pectin from apple were purchased from Sigma-Aldrich, USA. Pharmaceutical grade glycerol and allantoin were purchased from La Corona, S.A. de C.V., México, and Droguería Cosmopolita, S.A. de C.V., Mexico.

### 2.2. Preparation of the Hydrogel

For the elaboration of the hydrogels, 5% polymeric solution of pectin was prepared and physically crosslinked with glutaraldehyde (10^−3^ M) in acid medium [20]. The solution was mixed under constant stirring by 2 hours at 35°C. Then, the hydrogel solution was enriched with allantoin (to obtain PA hydrogel) in 1 : 1 relation with respect to pectin grams used. Finally, the solution was plasticized with glycerol in 1 : 1 relation with grams of pectin used, under continuous stirring for 1 h at 35°C. At the end of the mixing, the solution (10 ml) was poured in disposable polystyrene Petri dishes of 113 cm^3^ and then dried at ambient room temperature to form air-dried films.

### 2.3. Characterization of the Hydrogel

The swelling studies of the hydrogels were conducted gravimetrically at room temperature using distilled water. Weights of dried and hydrated samples were registered as follows: the weight of dried hydrogel samples (5 mm × 5 mm) was determined, after which the samples were immersed in distilled water [20]. Finally, the samples were removed, and the weight of the hydrated film samples was calculated. The equilibrium swelling was determined by the equation (1).

(1) $$\begin{array}{c} \%\text{swelling}=\frac{\text{Ws}-\text{Wd}}{\text{Wd}}\times 100, \end{array}$$

where Wd is the weight of the dried hydrogel and Ws is the weight of swollen hydrogel. Five samples were used for each type of PA hydrogel. Six experiments were performed to calculate the swelling degree.

The instrumental analysis was performed as follows: Fourier transform of the infrared spectrum of absorption (FTIR spectra) of pectin and allantoin crosslinked films were reported using a Bruker SA, Alpha-T FTIR system at a wave number from 4000 to 400 cm^−1^.

The surface morphology of the film was examined by scanning electron microscopy (SEM) in a JEOL JSM-6010PLUS under low vacuum conditions from 3 kV to 15 kV using secondary electrons with magnifications of 200×.

Confocal laser scanning microscopy (CLSM) was employed to determine the distribution of allantoin in a Carl Zeiss LSM700 microscope, with a 488 nm laser line (green) and 555 nm lase line (red) of intensity; the samples were analyzed under dry and swollen conditions. The contact angle of the samples was calculated by the sessile drop method with an attention contact angle meter.

### 2.4. In Vivo Assay

For the *in vivo* assay, all the procedures were previously approved by Biotic Comity at the University (CIBE-2017-1-45). 27 female Wistar rats were used with a body weight around 200-240 g. Rats were placed in 43.2 × 34.0 × 19.8 cm polycarbonate cages for a mount at 21°C and 45% of humidity with pellet diet until required weight was reached [21–23].

#### 2.4.1. Surgical Procedure

For surgery, rats were anesthetized intramuscularly in the gluteal region of the right limb with xylazine (10 mg/kg) and tiletamine/zolazepam (30 mg/kg) [23–26]. For in vivo assay, surgical excision of 2 cm in diameter was made in dorsal area [25, 27]. For the experiments, 4 rats were tested at 4, 8, 15, and 21 days. In total, two groups of 16 rats were used (*n* = 16), 16 rats for group PA (90% All-PcH) and for group C. Control rats were used in each day time tested (*n* = 8): group C for negative control with no treatment and group PA treated with pectin and allantoin film. A film was placed in the dorsal area to cover the surgical excision made and replace it with a new film after the biodegrading of the film on the wet wound at 6, 18, 48, and 72 hours; for the rest of the experiment, the wound started to dry; then the film was replaced only in a few sections of the wound when it was necessary during the 8th day. For the 9th day, it was not necessary to place another PA film since the wound area was dry and healing. All experiments were performed according to the associated laws and institutional guidelines (human and animal welfare) and approved by Biotic Comity at the University (CIBE-2017-1-45).

#### 2.4.2. Determination of Wound Healing in Dorsal Area

Measurements of the surgical excision area were made. Rats were sedated in a chamber with isoflurane for 30 seconds. The difference in the area was registered at 4, 8, 15, and 21 days compared with that in the size area at day 0 to calculate the reduction of the excision area using the following formula:

(2) $$\begin{array}{c} \%=\left({\text{AD}}_{0}-{\text{AD}}_{t}\right)\frac{100}{{\text{AD}}_{0}}, \end{array}$$

where AD_0_ is the excision area at day 0, AD_*t*_ is the excision area at day “*t*,” and “*t*” represents days 4, 8, 15, and 21.

## 3. Results

### 3.1. Physical Crosslinking of Pectin

The optimum stirring speed was 50 rpm since a homogeneous mix of the polymer was obtained. Stirring time was established at 2 hours, until the stirring speed was the lowest within the experimental matrix thus to get a homogeneous mix, without the foam presence. The adequate drying temperature was set at room temperature for 5 days to assure a uniform drying pattern of the hydrogel.

### 3.2. Plasticization with Glycerol and Incorporation of Allantoin

**Figure 1:**
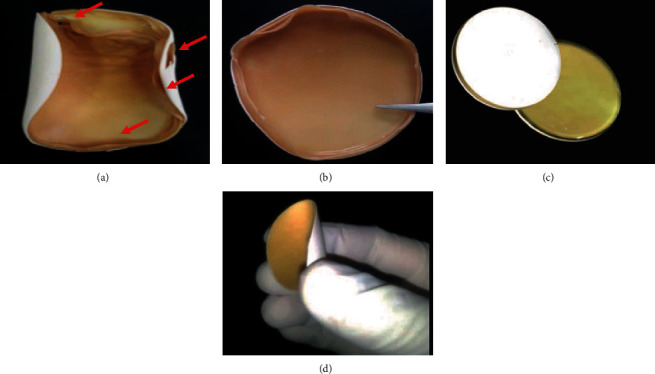
(a) Pectin hydrogel enriched with allantoin without glycerol: red arrows indicate film fractures in both sides. (b) Pectin hydrogel enriched with allantoin plasticized with glycerol. Pectin hydrogel enriched with allantoin: (c) white side rich in allantoin and brown side rich in pectin and (d) more flexibility of the film due to glycerol.

The obtained PA hydrogel film was brittle and easily broken and not suitable to cover a wound in a homogeneous way. In order to diminish this, glycerol was used as a plasticizer (Figure 1). The optimum concentration relation between pectin and glycerol was 1 : 1 to obtain a more flexible and handled PA hydrogel film (Figures 1(c) and 1(d)).

Moreover, it was found that the suitable allantoin concentration to enrich the hydrogel was 90 wt% and 100 wt% of the relation 1 : 1 with respect to pectin grams used. Indeed, more homogenous allantoin distribution among the hydrogels in the final product was observed using 90 wt% and 100w% of allantoin to prepare the film. Since these two concentrations of allantoin were established to be more suitable for the development of the hydrogel, the characterization was carried out for hydrogels containing 90 and 100 wt% allantoin in relation 1 : 1 with respect to the amount of pectin used (90% All-PcH and 100% All-PcH), respectively.

### 3.3. Swelling Kinetics

**Figure 2:**
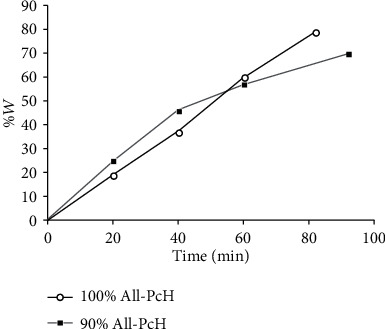
Swelling (%) as a function of time for pectin hydrogels with 90 wt% and 100 wt% allantoin concentrations in relation to pectin concentration. Result represents the mean (±s.e.m.) number of samples tested at 25°C to obtain a reliable value. Mean ± s.e.m. for *n* = 5 for each test (*p* < 0.05).

The maximum swelling degree for 90% All-PcH was 69.3%, and the measured samples could no longer hold water after 90 minutes after immersion in distilled water. On the other hand, 100% All-PcH had a maximum swelling of 79.35%, and the measured samples could no longer hold water after 80 minutes after immersion, as observed in Figure 2. This shows that, at a higher concentration of allantoin, the hydrogel has a higher sorption capacity, which was expected since the allantoin has hydrophilic functional groups [7], nano and microporous in its structure increasing the adsorbing area of the film [19].

### 3.4. FTIR

**Figure 3:**
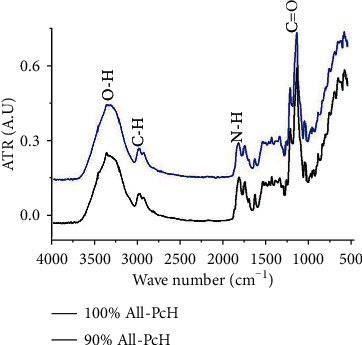
FTIR for pectin hydrogels with 90 wt% All-PcH and 100 wt% All-PcH.

Structural validation of the physical crosslinked was confirmed as shown in Figure 3 for each PcH with 90 and 100 wt% allantoin concentrations, where it is clear both hydrogels do not exhibit any chemical interaction among its components. Dominant pectin and glycerol peaks from 3400 to 3200 cm^−1^ are assigned to stretch vibrations of the OH bonds, and those from 2938 to 2880 cm^−1^ are related to stretches of the CH bonds asymmetrically and symmetrically, respectively, and also 3 peaks correspond to allantoin at 1714, 1661, and 1606 cm^−1^ from bending vibrations of the amide NH group.

### 3.5. Contact Angle

**Figure 4:**
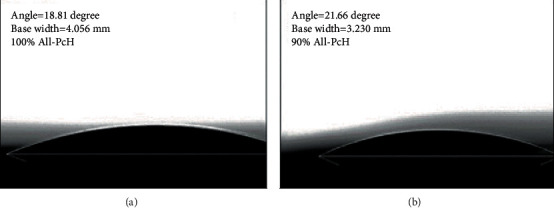
Contact angle for pectin hydrogels with (a) 100 wt% All-PcH and (b) 90 wt% All-PcH allantoin concentrations.

Figure 4 shows the contact angle for the 90 wt% All-PcH as 21.86°, while 100 wt% All-PcH was 18.81°. The concentration with 100 wt% allantoin was more permeable given that allantoin is a hydrophilic compound with functional groups predominantly polar [7] even the presence of the glycerol (a hydrophobic component); this is an advantage for the hydrogel practical use, since it guarantees the adhesion to a wound as long as it is humid, allowing allantoin to be in immediate contact with the system of growth factors, fibroblasts, and inflammatory cells during the process of tissue regeneration.

### 3.6. Morphology (SEM)

**Figure 5:**
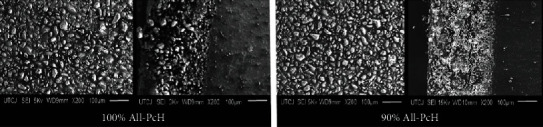
SEM micrographs for pectin hydrogels with 100 wt% All-PcH and 90 wt% All-PcH.

Figure 5 for 100 wt% All-PcH shows a micrograph of the longitudinal area for the hydrogel rich in allantoin, presenting uniform distribution of allantoin with granulate shape forms ranging in the size of 1.2 *μ*m to 54 *μ*m; furthermore, a cross section of the same hydrogel sample shows a layer rich in allantoin within a thickness of 300 *μ*m and incrustations of active ingredient can be also noted within the pectin rich layer. On the other hand, 90 wt% All-PcH the micrograph of the longitudinal area shows distribution of the allantoin; however, on the cross section, the layer rich in allantoin is slightly thinner measuring 281 *μ*m and the incrustation of allantoin due to the layer rich in pectin is less concentrated in contrast with the 100 wt% All-PcH. Having two well-differentiated layers in the hydrogel is an advantage because it can suggest that once the layer rich in allantoin is applies directly to the wound, it will have an immediate releasing action of the active ingredient while the layer rich in pectin will continue to retain the active ingredient and deliver it at a lower concentration.

### 3.7. Morphology (CSLM)

**Figure 6:**
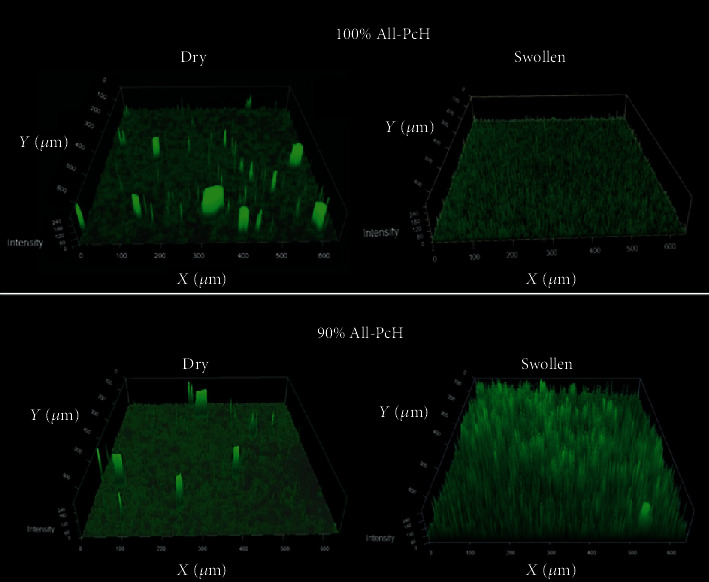
CSLM-10× magnification micrographs for pectin hydrogels with 90 wt% All-PcH and 100 wt% All-PcH.

To determine allantoin distribution inside the hydrogel, dry, and swollen conditions, images were captured for each concentration of allantoin.  Figure 6 shows 90 wt% All-PcH and 100 wt% All-PcH samples, in which allantoin is settled at the base, as well as compacted in peaks under dry conditions; meanwhile, at swollen conditions, the peaks become narrow and allantoin was distributed more uniformly among the hydrogel. These results suggest that once hydrogel is in contact with the wound, the allantoin will be distributed more evenly as long as there is moisture in the wound.

### 3.8. In Vivo Assay

**Figure 7:**
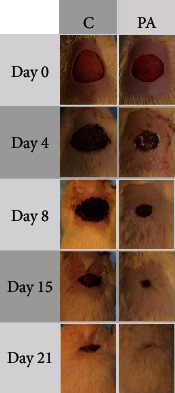
Images of wound in the dorsal area during the healing process.

After all these results, for the in vivo assay only, 90 wt% All-PcH sample was used because this film contains the maximum concentration of allantoin recommendation of FDA [28]. In vivo assay of 90 wt% All-PcH film showed remarkable diminish of the time healing process. Excision area reduction of groups C and PA is shown in Table 1 and Figure 7. Higher size area excision reduction was observed in group PA compared to group C. Complete healing of excision was observed at day 18 in group PA and at day 21 in group C.

## 4. Discussion

This study determines that the PA film demonstrates to have adequate surface and biocompatible properties as well as good wound healing promotion from an in vivo test. Moreover, the use of the PA film in animal tests for only 8 days supports the application to promote wound healing in medical applications.

It was observed the adequate concentration of allantoin was 90 wt% showing maximum swelling values were around 60 to 70 wt%. It is well known that sorption capacity is important to provide a humidity environment and promote a healing process. This tendency was also confirmed by contact angle measurements. Contact angle values slightly decrease with the increment of allantoin content in the film. Contact angle values show hydrophobicity of the surface, reported as a key point for tissue regeneration. Protein adsorption is important for the healing process and cell proliferation. In this case, the PA film provides a suitable environment for growth and clotting factors, as well as inflammatory cells essential for wound healing.

SEM and CSLM images showed uniform distribution of allantoin in the PA film. Furthermore, two well-differentiated allantoin and pectin layers. Allantoin is settled at the base and well distributed along the hydrogel film. This could permit allantoin distribution among wound areas. The pectin layer will retain and deliver the active ingredient allantoin allowing direct contact to wound helping to the healing process.

Incisional and excisional wounds are the two models that allow the healing process of cutaneous wounds, as reported by Giusto et al. (2017). In this study, the contraction rate of wounds treated with the PA film was macroscopically evaluated. The results demonstrated that the PA film accelerates wound healing showing significant size reduction in the wound dorsal area in less time compared with control. In addition, at day 4, the contraction rate higher than the control was observed. A more significant difference was observed after day 8, showing a positive effect on the healing process.

Moreover, for further applications of the PA film in medicine, fibroblast adhesion to hydrogel surface should be considered. Although this study showed acceptable physical, microscopy, and biological properties of PA films, still, research using other parameters and biologic conditions must be performed. However, we combined by the first time two natural ingredients reported to speed up the healing process to develop a new biomaterial for the alternative treatment of skin injuries promoting healthy tissue renewal.

## 5. Conclusions

It is possible to obtain a pectin gel plasticized with glycerol and enriched with allantoin. Characterization by infrared spectroscopy demonstrates that a hydrogel of pectin and allantoin was obtained, although peaks of pectin and glycerol hold sway in the spectra of the hydrogels than peaks corresponding to the amine and amide groups of the allantoin. The characterization by SEM and CSLM revealed that there is an even distribution of allantoin, both in the surface and in the inner membrane of the hydrogel, for both concentrations of allantoin. So far, it can be said that the pectin-coated hydrogel with 100 wt% allantoin is the best candidate for the next phase of the project, since, according to the contact angle, it proposes a stronger adhesion to the wound. In vivo assay results showed remarkable reduction of wound area by using PA hydrogel from day 4 and the complete healing process at day 18. PA hydrogel reduces the total healing time by 25% approximately. These results indicated that PA hydrogel films could become the leading biomaterial candidates for wound healing treatments.

## Figures and Tables

**Table 1:** Percentage size reduction of the surgical excision wounds in dorsal areas during the healing process.

*t* (days)	C (%)	PA (%)	Significance
4	18.48 ± 3.12	22.26 ± 9.38	0.28
8	47.49 ± 3.89^a^	69.99 ± 6.02^b^	0.006
15	83.99 ± 2.15^a^	90.44 ± 2.90^b^	0.018
21	93.58 ± 3.70^a^	100^b^	0.048

LSD (least Significance difference) Test (*n* = 16for each group test (*p* < 0.05)).

## Data Availability

All data obtained from this study can be found in the research archives of the Master Program in Chemistry-Biology Science of the Autonomous University of Ciudad Juarez and can be requested through the corresponding author.
